# Comprehensive right heart systolic function assessment using cardiac magnetic resonance imaging after inferior ST elevation myocardial infarction

**DOI:** 10.1186/1532-429X-17-S1-P158

**Published:** 2015-02-03

**Authors:** Nor Hanim Mohd Amin, Seng Keong Chua, Chi Yen Voon, Asri Said, Ning Zan Khiew, Sian Kong Tan, Alan Fong, Tiong Kiam Ong

**Affiliations:** Cardiology, Sarawak General Hospital Heart Centre, Kota Samarahan, Malaysia

## Background

Acute Inferior ST Elevation Myocardial Infarction (Ac-Inf-STEMI) and associated posterior and right ventricular (RV) infarction has been extensively studied with electrophysiology and echocardiography. A recent CMR study showed that poor RV function was associated with poor long term survival post myocardial infarction. However, limited CMR data exists especially on short term clinical outcomes in Ac-Inf-STEMI.

## Objectives

Determining the RVEF, right atrial ejection fraction (RAEF), and other CMR characteristics in Ac-Inf-STEMI and association with clinical outcomes.

## Methods

53 patients presenting with Ac-Inf-STEMI by ECG, suitable for CMR, were recruited from August 2012 till April 2014. Most had thrombolysis followed by early routine invasive strategy while 2 had primary angioplasty. CMR was done during index admission and at 5th month.

## Results

Mean age was 53 ± 8.7 years, 90.6% males and 70% smokers. 45% had multivessel disease with 47 had culprit right coronary artery. Mean admission SBP was 98.9 ± 29.7 mmHg, DBP 60 ± 19 mmHg and HR 54 ± 16 bpm. 8 required atropine and 2 temporary pacing.

CMR detected 49 and 14 patients with inferobasal and RV infarcts while ECG diagnosed 26 and 30 patients respectively with corresponding posterior and RV infarcts.

Admission RAEF correlated with RVEF (p <0.0001 r=0.479), and RVEF with LVEF (p< 0.001, r=0.49). Left ventricular infarct size correlated with LVEF (p=0.04, r=-0.28). RVEF improved at month 5 (Week 0 mean RVEF = 55.9%, Month 5 mean RVEF = 60%, p=0.022). LVEF improved from 49.6% to 52.5% (p<0.001) but RAEF (48.8 vs 50.3%, p=0.16) did not. In patients without RV infarction by CMR or ECG criteria, LVEF still showed signifcant improvement (p= 0.003), while RAEF and RVEF did not. 2 early mortalities (within 2 weeks) had triple vessel disease, poor LVEF and RVEF < 40% and extensive microvascular obstruction. 2 late mortalities (month 5) had single vessel disease, reduced LVEF, but minimal MVO and normal right heart systolic function. None who survived had all of the above adverse prognosticators.

## Conclusions

Our study demonstrated interdependence between right atrial with biventricular systolic function post Ac-Inf-STEMI, and significant recovery of RV systolic function 5 months after a CMR or ECG-confirmed RV infarction. Clinical and CMR characteristics in 2 observed early mortalities showed that the CMR evaluation in these patients may have additional short term prognostic value. This study suggests that comprehensive right heart systolic function assessment with CMR may not be unreasonable in patients presenting with Ac-Inf-STEMI.

## Funding

Self-funded.

